# A surrogate safety analysis at sharp gore areas of diverging freeway ramps using micro simulation under congested traffic conditions

**DOI:** 10.1038/s41598-023-49728-4

**Published:** 2023-12-18

**Authors:** Abdulaziz H. Alshehri, Muhammad Umer Farooq, Abdul Farhan, Ahmed M. Yosri, Fayez Alanazi, Ahmed Farouk Deifallah, Mohamed Ahmed Okail

**Affiliations:** 1 Department of Civil Engineering, College of Engineering, Najran University, 66446 Najran, Saudi Arabia; 2 Civil and Environmental Engineering Department, University of Nebraska – Lincoln, Lincoln, USA; 3 Civil Engineering Department, Delta University for Science and Technology, Belkas, Egypt; 4 Present Address: Civil Engineering Department, College of Engineering, Jouf University, Sakakah, Saudi Arabia; 5 Structural Engineering and Construction Managment Department, Future University in Egypt, New Cairo, 11835 Egypt; 6 Faculty of Engineering and Technology, Badr University in Cairo (BUC), Cairo, 11829 Egypt

**Keywords:** Civil engineering, Engineering, Environmental sciences

## Abstract

Safety at the gore areas near diverging ramps is very crucial during planning and implementation of highway safety improvement programs. Limited research has been conducted on safety at the gore areas on arterial roads. This study aims at investigating the impact of improving a sharp gore area in Lincoln, Nebraska by performing a micro simulation before-and-after study with respect to its underlying state of safety and congestion. Data on travel times and traffic volumes for peak hours are incorporated after successful calibration to find out how a geometric intervention can decrease mobility issues as well as the likelihood of crash involvement. This study has utilized VISSIM software package to run the simulation however, to perform safety analysis, Surrogate Safety Assessment Model (SSAM) is used which is developed by the Federal Highway Administration (FHWA). During the model calibration, custom driving behaviors are created to represent driving tendencies of familiar drivers. The simulation results indicated that by adding an auxiliary lane near the gore area, the mobility issues such as bottle necks, lane changing dilemmas and queue lengths are substantially decreased. However, geometric interventions such as provision of a separate lane, increasing ramp spacing, nose spacing, deceleration area and queue storage area considerably reduced the likelihood of rear-end and lane changing crashes. Surrogate safety assessment in diverging ramps, particularly for sharp gores, has not previously been studied, and this study can serve as a primary footmark for future research on ramp-gores safety.

## Introduction

The safe operation of on and off-ramps is very critical for the efficient operation of the traffic network. Congestion at one of the ‘on’ or ‘off’ ramps will have a ripple effect, causing mobility issues throughout the network^1–3^. Several times, congestion issues such as bottlenecks or lane-changing dilemmas during peak hours can affect driving abilities and cause severe crashes^4^. These crashes not only pose a difficult challenge to transportation agencies, but they also have a significant economic impact^5,6^.

Identifying and analyzing the safety efficacy of various transportation facilities is an important area of road safety research. Typically, past crash data are used to evaluate factors that cause traffic crashes on various sites, but this data collection process is costly and time consuming^7,8^. Researchers must sometimes wait for long periods of time and conduct impact studies to see if there will be any crashes in their case studies. However, for the areas where agencies look into new designs, the conventional method cannot be used as it’s not possible to record sizable amount of past crash data and agencies are reluctant to perform experimental public studies by creating pilot studies. Under these conditions, the researchers face setbacks in evaluating the safety situation for their intended study locations since they have to wait for the collision events to occur which is unwanted. To cater for this gap in the road safety analysis, the Surrogate Safety Assessment Model (SSAM) developed by FHWA comes into play and replaces the need of having big past crash datasets with collision observation of real-time driving conditions^9^.

In this study, VISSIM is used along with the Surrogate Safety Assessment Model (SSAM) to evaluate the current operational safety of an on ramp situated on N Antelope Valley Pkwy, which is a local two-lane on each side road in Lincoln, Nebraska. The study started off with data collection of travel times, queue lengths, and physical elements of on-ramp such as lane width, shoulder width etc., and used the collected data to create a pre-intervention simulation model in VISSIM. After model calibration, a few geometric elements of the onsite conditions are changed such as adding an auxiliary lane and increasing ramp spacing in the post-intervention simulation model. Furthermore, the before and after trajectory data are analyzed in SSAM to estimate the total number of rear-end, crossing, and lane-changing crashes that are likely to occur near the gore area of the off-ramp. This paper will contribute to understanding whether SSAM is useful for analyzing expected crashes at gore areas of diverging ramps, particularly for sharp gores with congested traffic flow conditions, which have not previously been studied and will eventually serve as a primary footmark for future ramp-gore safety research.

The structure of the rest of this paper is as follows. The next section provides a review of pertinent literature followed by a section describing the methodology and data collection. The ensuing section describes the simulation and Surrogate Safety Analysis followed by results. The last section concludes the paper by summarizing major findings and providing key insights obtained from the research.

## Literature review

When ramps become congested, traffic on arterial roads and freeways begins to crawl. Some ramps are extremely close to one another, resulting in longer travel times, frequent queues, and crash hazards^5^. Also, due to the lack of a conflict point near the ramps, strict management and control measures are not generally implemented^7^. As a result of traffic volume conflicts near on and off ramps, freeways and local roads become congested^10^. Leisch^11^ investigated the spacing of urban expressway interchanges. Al-Kaisy et al.^12^ thoroughly investigated the relationship between ramp traffic flow, traffic capacity, main road traffic flow, length of deceleration lane, and lane number. Munoz and Daganzo^13^ provided a detailed description of the traffic operation of the bottleneck section of the highway exit ramps. Furthermore, Jayakrishnan et al.^14^ simulated urban road traffic flow and proposed that there are many differences between urban road and highway traffic flow, particularly the mutual influences of multiple vehicle types on urban roads. Kojima et al.^15^ used an x-shaped crossroad as the simulation object to investigate the mixed behavior of individual vehicles and investigated specific areas of the diverging section that are affected mostly in the events of a crash. The frequency of changing lane behaviors and braking behaviors were identified as the main determinants of traffic crashes in diverging sections.

There are three main approaches used to evaluate the safety condition of any road facility, which are usually based on short- and long-term safety analysis. (a) Using crash frequency by studying past crashes, (b) Indirect analysis of safety measures and (c) Safety analysis by statistical investigation. The term "indirect safety measures" refers to the traffic conflict technique (TCT), which does not require crash data to be analyzed^16^. TCT has become popular for safety analysis in recent years due to the difficulties in collecting crash data due to a lack of detailed and comprehensive data. Perkins and Harris^17^ were the first to formally apply TCT in General Motors Corporation. Their strategy was to observe and count situations in which vehicles perform evasive maneuvers to avoid collisions. But because of human errors and limitations in addressing driving behavior, using this approach entailed flaws. Therefore, to meet the needs of research in crash evaluation, Surrogate Safety Analysis Model (SSAM) was introduced which is a pack of tools that detect different types of traffic conflicts based on real time data^18–26^.

With the help of SSAM, three main tasks can be fulfilled; (a) calculation of likelihood of a future crash and (b) computing the outcomes of an anticipated crash (c) finding the type of crash that will occur due to conflicts (rear-end, lane changing, cross-angled etc.) However, recently, many vehicle manufacturers have attempted to develop self-driving vehicles using artificial intelligence. These vehicles are equipped with a specialized collision avoidance system (CAS) that aids in the detection of any future collision or risk of crash involvement. These systems also use SSAM to calculate the likelihood of a future crash and warn the driver before its occurrence^23^.

Furthermore, St-Aubin et al.^24^ presented a methodology for automated conflict analysis at highway ramps by using before and after cross-sectional data. The proposed methodology was based on automated trajectory collection and behavioral analysis from surrogate safety measures. A simple highway section at on-ramps and off-ramps was studied to study the effectiveness of a lane-change ban treatment in Montreal, Canada. Video data were collected using a traffic surveillance system and mobile video camera unit. The study revealed that the treatment did not have a statistically significant impact on time-to-collision distribution. In addition, a recent study by Gruden et al.^26^ evaluated a confined pedestrian ramp of an old bridge under different flow conditions. Safety assessment was evaluated by safety surrogate measures and trajectories of individuals were recorded through trajectory tools. The effects of induced pedestrian mobility on the bridge in terms of safety were studied with the aim of preventing negative scenarios that could lead to a bad infrastructural level of service. Although there are many studies related to surrogate safety analysis in the literature, specific studies on gore areas of diverging ramps are limited and require further investigation^23–30,32,33^.

**Figure 1:**
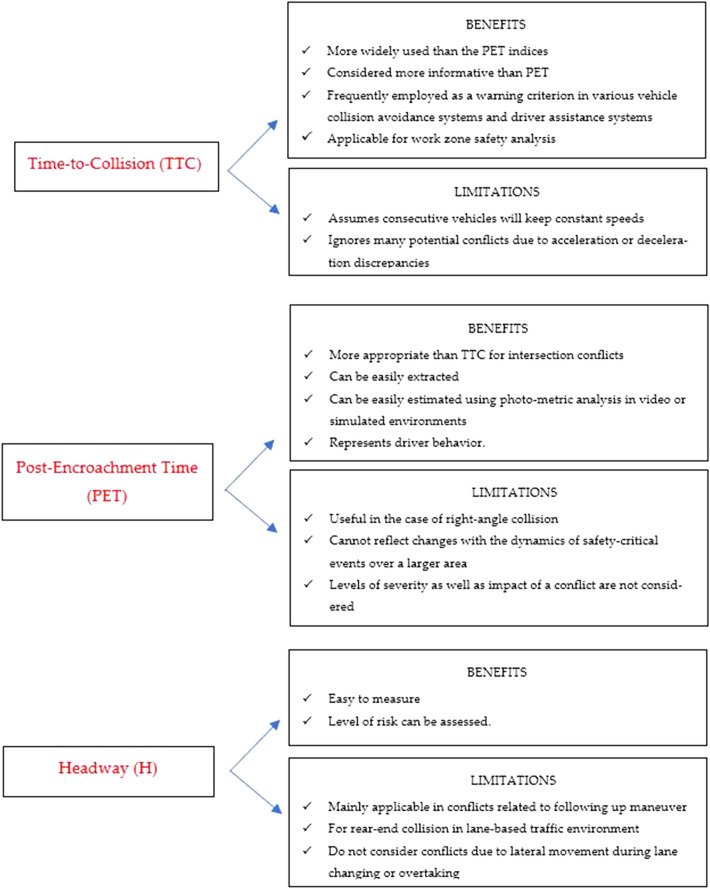
Limitations and benefits of simulation-based conflict indicators.

Table 1 outlines the application of various indicators to determine the potential conflict risk in a simulation-based approach. Furthermore, Figure 1 presents limitations and benefits of different simulation-based conflict indicators. The table lists various surrogate indicators, which embody the temporal and spatial attributes of unsafe vehicular interactions, along with their respective advantages and limitations.

**Table 1:** Pre-intervention queue lengths in feet.

Queue length (ft)	DCP-I	DCP-II	DCP-III
Average	73.5	64.2	26.7
Standard deviation	19.1	11.8	13.0
Minimum	68.0	57.0	23.9
Maximum	97.5	87.2	36.4

## Methodology

### Study area

**Figure 2:**
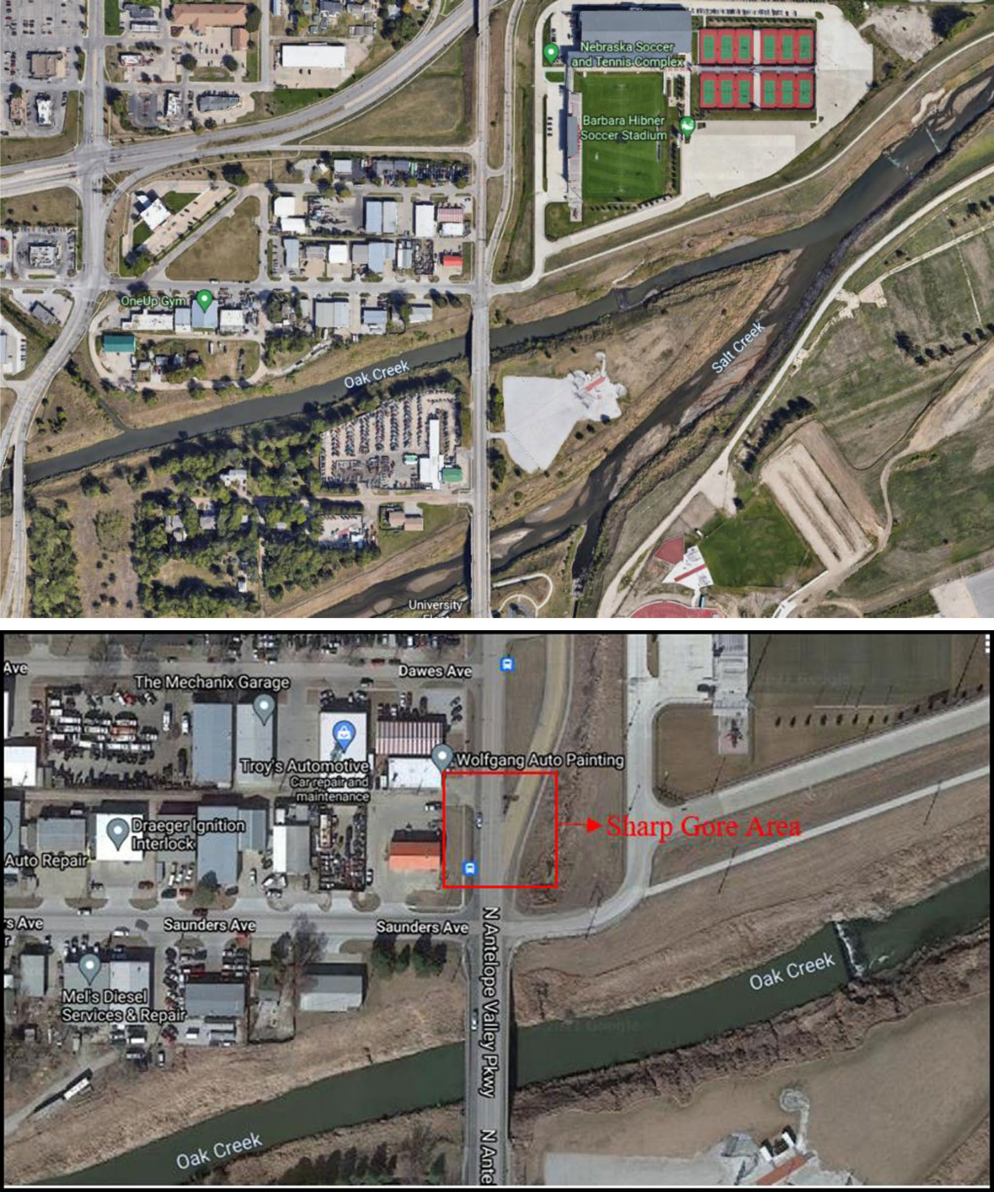
Overall network and study location showing diverging ramp with a sharp gore area (Source: maps.google.com).

This research utilized a case study of a sharp gore at a diverging exit ramp on N Antelope Valley Parkway in Lincoln, Nebraska, to examine the efficacy of SSAM in predicting traffic crashes at sharp gore areas near diverging ramps (Figure 2). The population density in Lincoln is 2,937.67 residents per square mile. The study area is located in Lancaster County, which is closely situated near the University of Nebraska and the Nebraska State Capitol. With a recorded Annual Daily Traffic (ADT) of 29,790 in 2018, this road network has one of the highest traffic counts in the county. Lancaster County, with a total population of 250,291 people, 99,187 households, and 60,702 families, is the second most populous county in Nebraska. The area is traversed by two major roads, Antelope Highway (where the location of the study area exists) and O Street, along with several regional roads running parallel to these main roads. According to the Nebraska Department of Transportation, from 2015 to 2020, there were a total of 74 crashes recorded of varying severity on Antelope Highway, highlighting the need for safety improvement studies on the network^31^.

### Ratinale of choosing the study area

**Figure 3:**
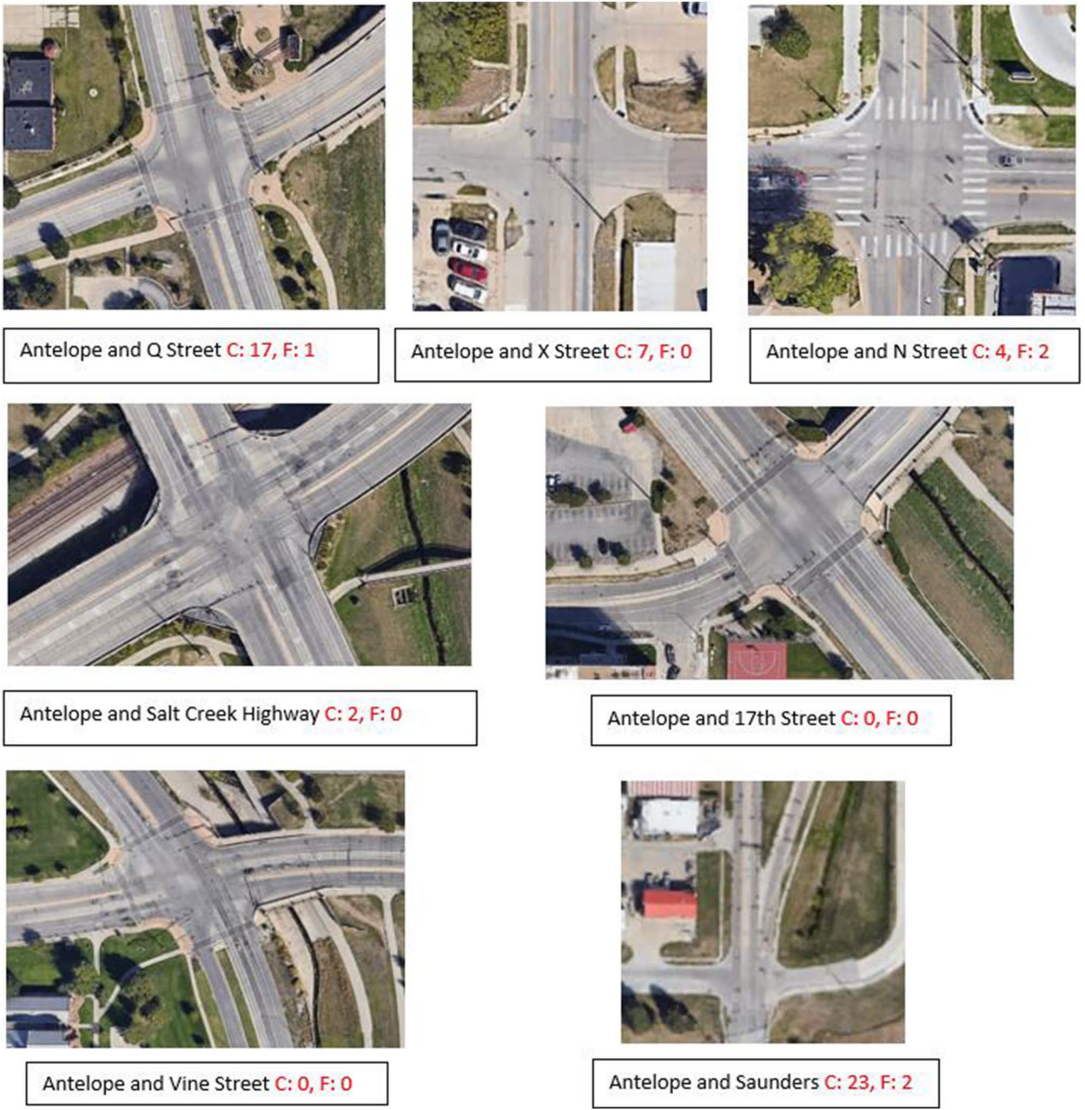
Crash (C) and fatality (F) count on major intersections on study route (Source: maps.google.com).

Within the confines of the county, there are seven major intersections along Antelope Highway that feature ramps. Our choice of study location was driven by the fact that it recorded the highest number of traffic crashes ranging in severity from 2015 to 2020, tragically resulting in two fatalities^31^. As depicted in Figure 3, the intersections along Antelope Highway and the corresponding total number of crashes and fatalities over the five-year span (2015–2020) are shown.

It is vital to clarify that in our analysis, we excluded crashes from consideration if their primary causes could be attributed to factors such as DUI, extreme weather, vehicle defects, reckless driving, or driver errors. Our focus was solely on incidents where inadequate signage, markings, or road geometry played a significant role in the occurrence of the reported crashes on major intersections on the study route.

### Simulation

During periods of heavy traffic, the preliminary on-site investigation revealed that drivers experienced confusion while diverging as well as driving straight. It appeared that the drivers are moving on a two-lane on each side road and just before the beginning of the gore, the two lanes converged into a single lane, resulting in a dilemma situation among drivers. In addition, the study area also persisted bottlenecks, long queue lengths, lane-changing dilemmas, and a higher risk of rear-end and lane-change crashes. According to AASHTO or NCHRP Report (687)^22^, which has standard guidelines for ramp and interchanges spacing, an examination of geometric features revealed that the gore area in the study has insufficient deceleration influence area, no white raised pavement markers, white and yellow post delineators, or sufficient nose area^27^.

Further visual inspection revealed that the sharp gore in the study had only an object marker, which is insufficient for the safety and mobility of traffic through this area, particularly during times of high volume and low visibility (such as heavy rains and at night).

**Figure 4:**
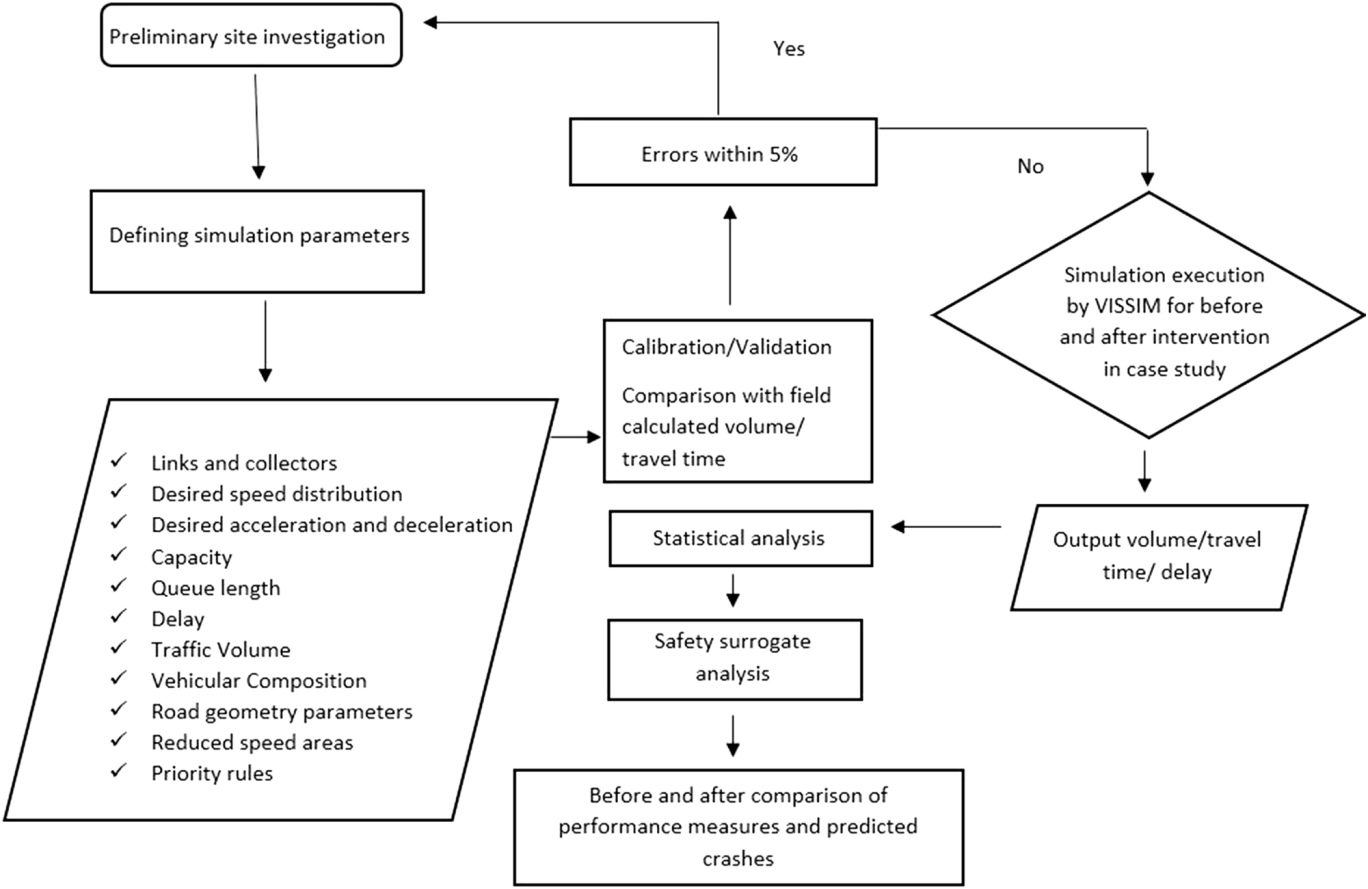
Research framework.

Figure 4 shows the research framework and gives insights into what parameters were initially recorded for model development in VISSIM. The primary task of the study methodology was to first analyze the current state of safety and congestion at the Gore, as well as on-site geometric and traffic parameters. The geometrics and physical properties of the gore are then changed in the simulation model to see if traffic congestion and delays were reduced. Later, performance measures of remedial actions for before and after intervention scenarios were evaluated by using statistical analysis to test the effectiveness of the simulated model. In the last step, pre- and post-intervention data output from VISSIM are used to predict future crashes by using SSAM.

**Figure 5:**
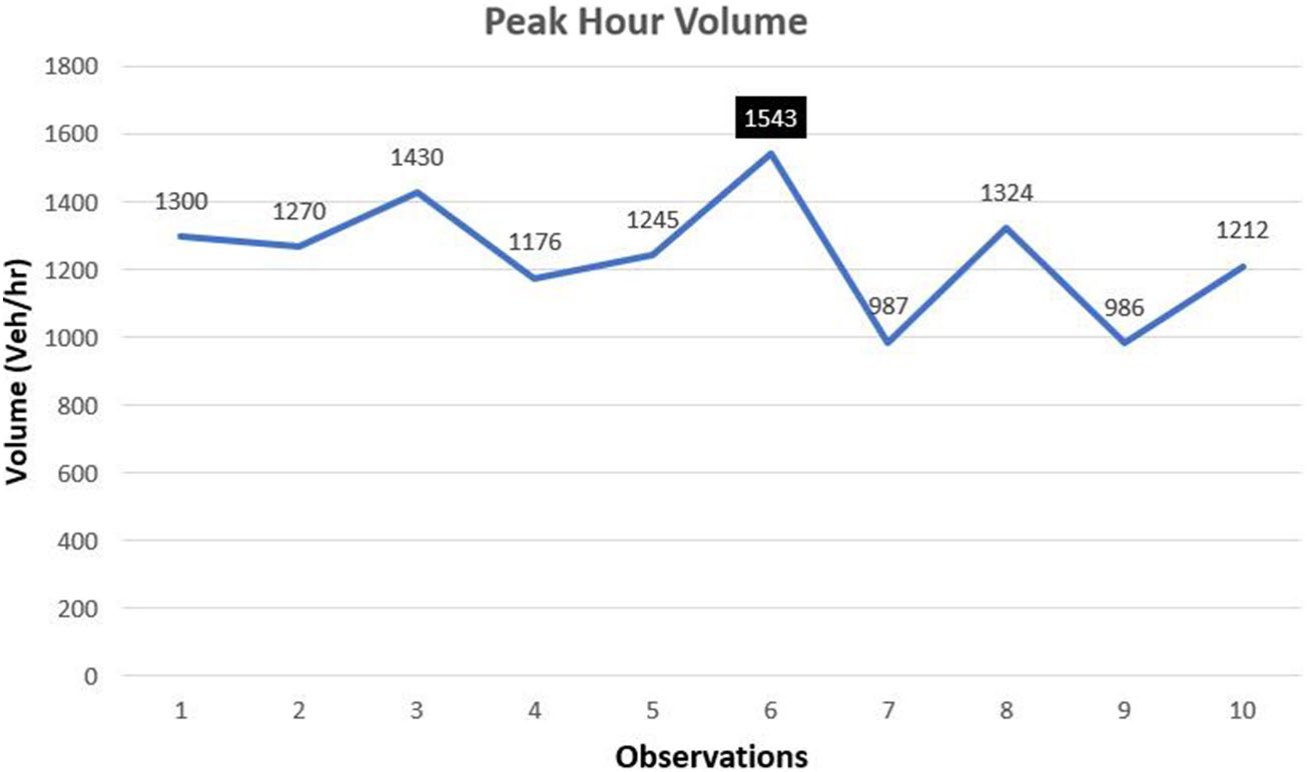
Manually recorded peak-hour volumes (veh/hr).

**Figure 6:**
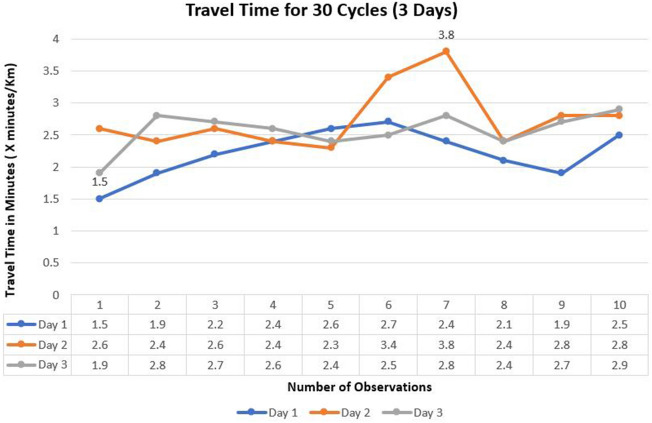
Manually recorded peak-hour volumes (veh/hr).

For model calibration, manual travel time data were recorded 30 times and then weighted averages of travel time were estimated. Figures 5 and 6 depict information about travel time and peak hour volume data. To obtain data pertaining to travel time, observations were conducted over the course of three days, with data recorded ten times each day. These observations were concentrated during peak hours, specifically between 8 AM and 10 AM and again from 4 PM to 6 PM. The highest recorded travel time was 3.8 minutes/km, and the lowest was 1.5 minutes/km. An average value of 2.5 min/km was used for calibration, and the field values in Figure 3 were compared to the travel time values generated by running the simulation in VISSIM. If the difference between the simulated and onsite values was greater than 5%, the process was repeated, and more trials were run in VISSIM until the error was reduced to 5% or less. Three trials were run during the calibration process to ensure the least amount of error. Figure 5 indicates that the highest peak hour volume recorded was 1543 vehicles/hr and the lowest was 986 vehicles/hr. The average value of approximately 1250 vehicles/hr was taken. However, peak hour volume was calculated using 15 minutes of manual counting.

The graph in Fig. 6 depicts general trends in peak hour volume data. In the present study, traffic volumes were calculated in vehicles/hr rather than vehicles/hr/lane because total volume values are required as input for each approach in simulation. Also, the other reason of taking volume in veh/hr was the reduction in the number of lanes from two to one on the main approach after the gore, which would have caused confusion and misestimation otherwise. Data points utilized for calibration have been a subject of significant discussion in existing literature. Several studies have supported the use of more data points for calibration^9,14,19^, while others have advocated for the use of fewer data points^28,29^. In our specific case, we opted to utilize 30 data points (collected in sets of 10 over three consecutive days) for the purpose of calibrating our model, based on practical considerations. In a prior study by Amirjamshidi^28^, a range of 2 to 10 data points were recorded for calibrating key parameters in microsimulation models. Furthermore, Dowling et al.^29^ provided a comprehensive explanation of their calibration procedures in their study. Their work highlighted the rationale behind the judicious use of a limited number of data points, specifically 10, in the calibration process. Dowling and their colleagues advocated a strategic approach that focused on identifying and fine-tuning the key parameters that exert the most significant influence on the model's performance. Subsequently, they extended their calibration efforts to encompass less critical parameters, effectively concluding the calibration process. This approach aimed to optimize calibration efficiency while minimizing the requirement for an extensive dataset for calibrating the model.

The second critical component of data collection was manually counting traffic during peak hours. Tally sheets were used for this purpose, and only vehicles traveling through or diverging by using the on-ramp towards the freeway from the arterial were counted. The reason for going to the site and manually counting the vehicles was a lack of updated AADT values for this road provided by the city of Lincoln, considering that the values were for 2016 and did not represent the current traffic volume. These manual counts were taken on three consecutive weekdays during peak hours.

**Figure 7:**
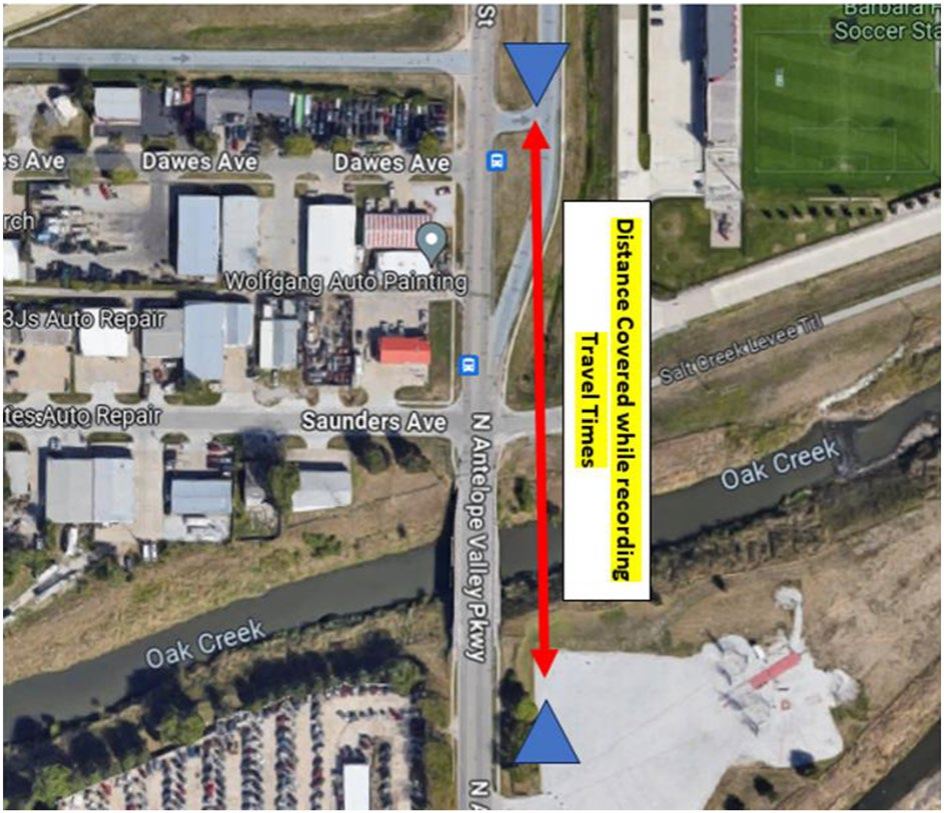
Distance covered while recording travel times.

**Figure 8:**
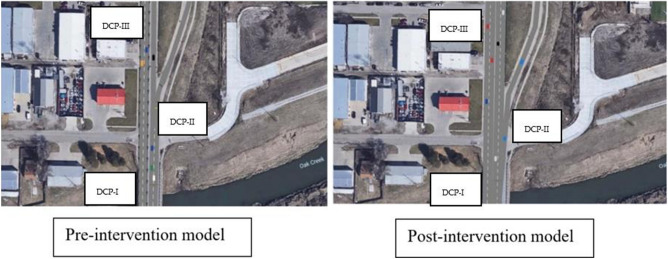
Pre- and Post-intervention models.

Figure 7 illustrates the distance for travel time collection. Figure 8 shows the links of the approach. For the study, three data collection points were taken that are referred to as DCP in the ensuing paragraphs. The first data collection point was a few feet apart from the inception of the 2-lane link, the second data collection point was just before the gore area where the pre-intervention model was estimating long queues and lane-changing dilemmas. The 3rd data collection point was just before the termination of the through-link which in the pre-intervention case has one link and has two lanes for the post-intervention scenario. A separate auxiliary lane is provided for the on-ramp so that the gore area can have sufficient space for declaration as well as queue storage.

It is imperative to emphasize our methodology for measuring queue length, which involved a manual approach. Initially, we conducted counts of vehicle passages at three specific locations denoted as DCP I, DCP II, and DCP III, as visualized in Figure 7. These counts were recorded in vehicles per minute consistently over a designated period. Subsequently, we tallied the number of vehicles entering the queue within the same timeframe. Leveraging this dataset, we applied Little's Law to calculate the queue length ^18^. To ascertain the average waiting time, we carefully observed randomly selected vehicles and documented their queue waiting durations.

It is essential to recognize that queue length is a multifaceted metric influenced by a variety of factors, encompassing the variability in vehicle arrivals, queue discharge rates, and the unique characteristics of the roadway^13,15,18^. In typical practice, advanced queuing theory models and traffic simulation software are employed to achieve precise and comprehensive queue length calculations. However, due to budget limitations that constrained this project, we were unable to employ these advanced tools. Consequently, the manual queue length calculation, while executed with precision, may not possess the same level of robust accuracy as it would with the utilization of advanced and more resource-intensive tools.

According to NCHRP report (687)^22^, a standard gore needs to have a sufficient deceleration zone and then a queue storage area. Considering that the pre-intervention ramp had insufficient deceleration space, more space is allotted for deceleration in the post-intervention scenario, with the provision of a separate auxiliary lane purely dedicated to on-ramp movements towards the freeway. In the post-intervention scenario, an auxiliary lane was provided as the study area had enough unused green space and lane addition can be easily accomplished.

### TTC and PET analysis and interpretation

**Figure 9:**
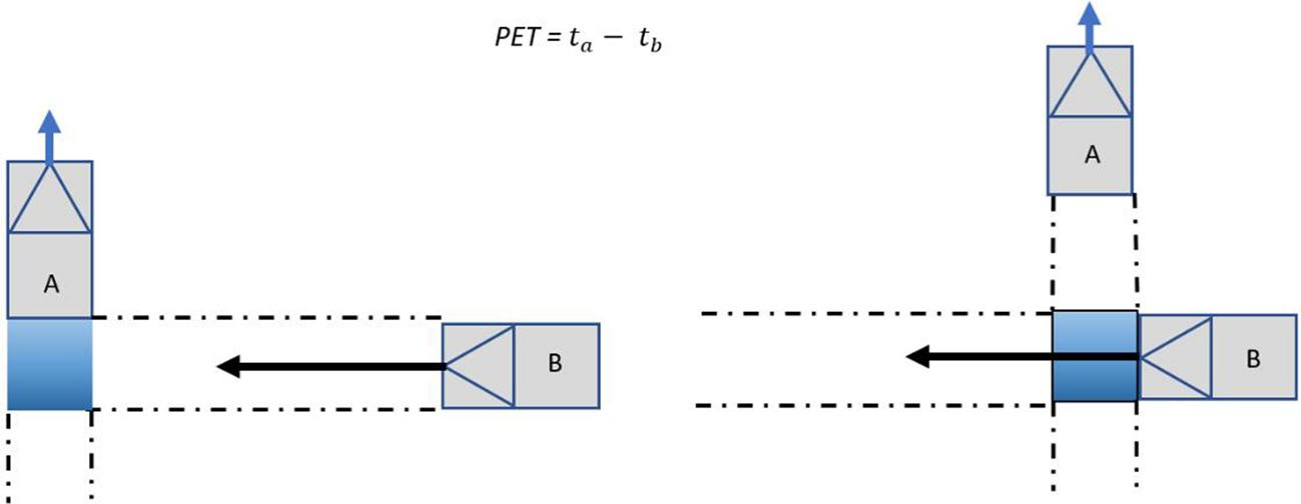
Diagrammatic representation of pre-encroachment time.

Two key parameters that the model use are Post-encroachment time (PET) and Time-to-collision (TTC). Post-encroachment time is the difference between the times that a vehicle enters a conflict point until another vehicle arrives at that point^19^. When PET values are smaller than the default threshold values in SSAM, there is a potential danger of collisions. Figure 9 shows details to understand pre-encroachment time.

However, TTC or time to collision is the time that remains until a collision occurs between two vehicles if the collision course and speed difference are maintained constant. Also, when TTC is smaller than the default threshold values in SSAM, there are potential dangers of collisions^16,20–25^. Furthermore, TTC is by far the most noteworthy surrogate measure of conflict severity, despite the fact that other surrogates (e.g., gap time, post-encroachment time, deceleration rate, etc.) have been implemented to quantify other aspects of conflict scenarios. Only a small amount of work has been utilized to customize or upgrade current general-purpose microscopic simulations to acquire conflict or other surrogate measures for various traffic situations, particularly for diverging exit ramps^24,25^.

**Figure 10:**
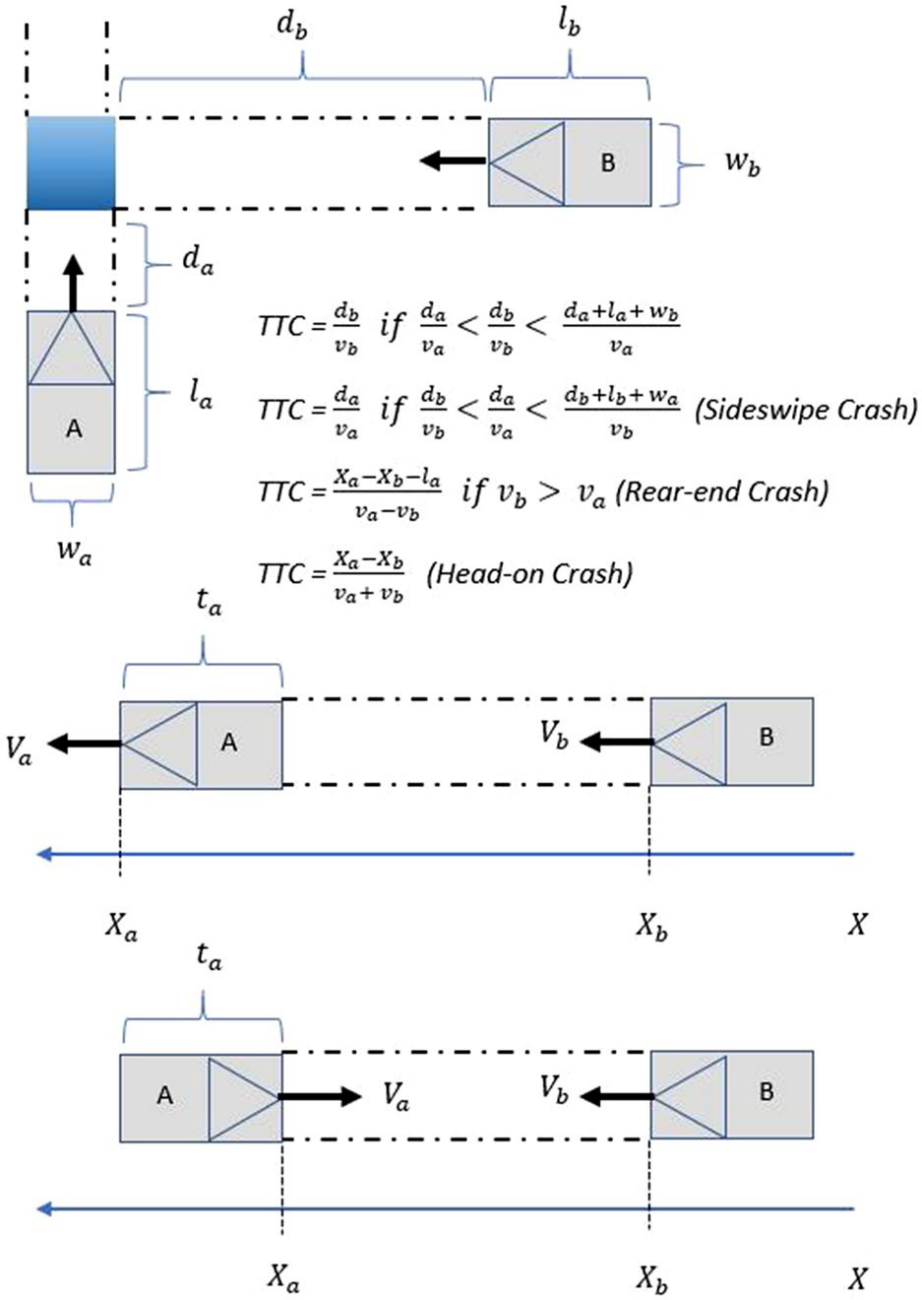
Diagrammatic representation of time-to-collision.

It is crucial to specify the methodology for estimating the probability of collision at any given time within the default SSAM model. To make the TTC measure helpful in the context of road safety, it is necessary to comprehend its association with collision likelihood, assuming that it exists reliably. Unfortunately, establishing a formal relationship between the two still necessitates extensive empirical and theoretical research from the driver behavior point of view. However, it is commonly recognized that spatial and temporal proximity tends to increase collision risk exposure, particularly when a collision course is involved. According to the SSAM model, considering that TTC is used to measure proximity in both space and time, it is suggested as a means of empirically assessing the likelihood of collision of an interaction at time *t,* over a time step $\Delta t$, taking into account TTC and other unspecified factors such as reaction times of drivers, conditions of visibility, performance of the vehicle, which is empirically incorporated and represented by $\theta$ (Fig. 10)^19^. Taking all these aspects, a relationship is formalized by default in the SSAM model:

1 $$PC\left(t\right)=f (TTC\left(t\right), \theta )$$

where $PC\left(t\right)$ is the probability of collision at any time (*t*) which depends on $TTC\left(t\right)$ and $\theta$. As defined for time-to-collision, the probability of a collision for a TTC of 0 is 1:.

2 $$PC\left(TTC\left(t\right)=0,\theta \right)=1$$

Since vehicles traverse at varying speeds, some degree of bias in the sample will exist as slower or faster vehicles are given more weight over time or travelled distance, respectively.

## Results

**Figure 11:**
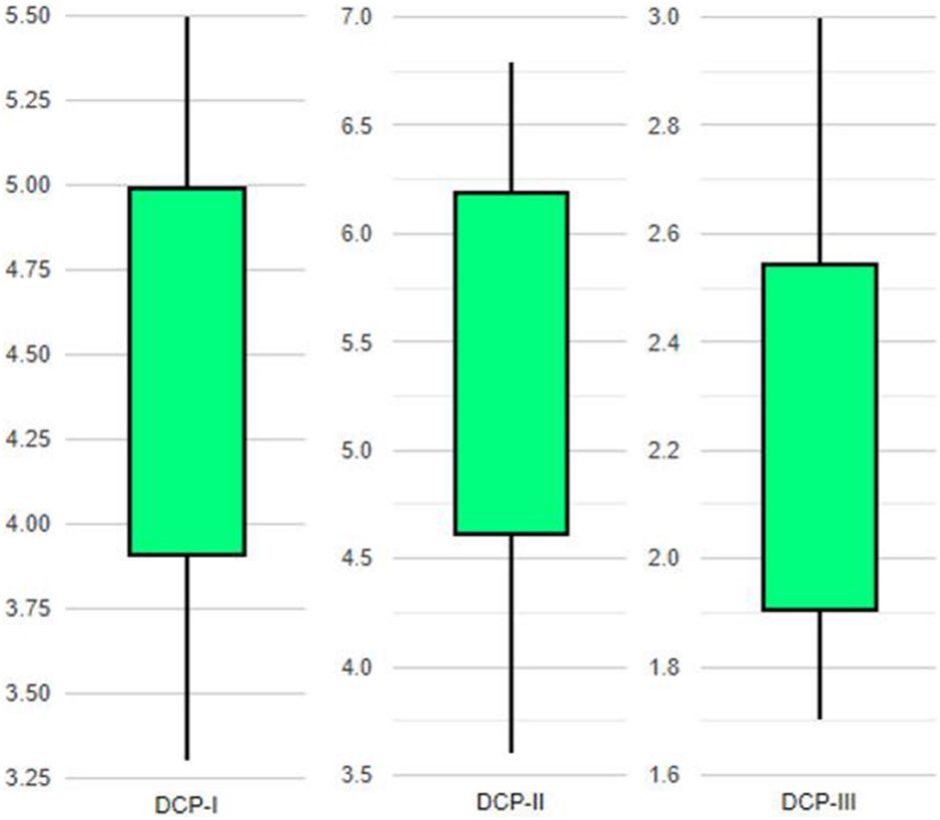
Boxplot showing the distribution of post-intervention queue lengths.

Table 1 and 2 give descriptions of mean values, standard deviations and minimum and maximum values for our data collection points 1, 2 and 3 for pre- and post-intervention simulation scenarios. Additionally, for post-intervention scenario boxplots are presented in Fig. 11 showing the distribution of post-intervention queue lengths.

**Table 2:** Post-intervention queue lengths in feet.

Queue length (ft)	DCP-I	DCP-II	DCP-III
Average	4.5	6.8	2.1
Standard deviation	0.7	0.6	0.7
Minimum	3.3	3.6	1.7
Maximum	5.5	5.6	3.0

It is noticeable that pre-intervention scenario has long queue lengths, the highest near the gore area. However, there is a drastic decrease in the queue lengths after a separate lane addition in the post-intervention table for queue lengths (Table 2). Table 3 gives details on t-testing and the total percent reduction in queue length values. A simple t-testing was performed to check the effectiveness of simulation model. Two conditions of hypothesis testing were considered.

**Table 3:** Results of a student t-test (Sample size = 20).

Data Collection Points (DCPs)	Average queue length (feet)			
	Pre- intervention	Post- intervention	Percent reduction	P-value
DCP-I	73.5	4.5	93.87%	0.004
DCP-II	64.2	6.8	89.40%	0.001
DCP-III	23.9	2.1	91.21%	0.001

${H}_{o}$: The queue length on DCPs for pre and post intervention is the same.

${H}_{1}$: The queue length on DCPs for pre and post intervention is different.

The results of statistical analysis revealed that for all three DCPs, the P-values are less than 0.005 (or are significant at 95% confidence interval). It rejects the null hypothesis indicating a significant difference between the queue lengths values for before and after scenario, demonstrating that the queue lengths have decreased considerably. For DCP-I, the highest percentage reduction of 93.87% was observed after adding an auxiliary lane in the post-intervention simulation. However, DCP-II and DCP-III indicated 89.40% and 91.21% percent reductions in average queue length for post-intervention scenarios, respectively. The highest percentage reduction in DCP-I can be attributed to the fact that this data collection point was located at the start of the 2-lane link, presumably causing the simulation to generate more volumes, resulting in more delays in the pre-intervention scenario. However, averaging queue lengths for all data collection points still results in a percentage reduction greater than 90%, which is significant.

The final important task of this study was to assess surrogate safety using the SSAM model. A few important SSAM parameters were obtained, such as the default angles for rear angle and crossing angle crashes, which were 30 and 80 degrees, respectively. By using SSAM’s built-in functions, the results from the analysis revealed that after adding a lane and improving the physical properties of the gore as shown on Table 4, there is a significant decrease in the likelihood of crash occurrence. Prior to lane addition, the vehicle trajectory data in SSAM predicted two lane changes and thirteen rear-end crashes; however, with lane addition and design improvements to the gore area, the number of crashes was reduced to only two.

**Table 4:** Total predicted crashes estimate by SSAM.

Type of crashes	Pre-Intervention	Post-Intervention
Unclassified crashes	0	0
Crossing crashes	0	0
Rear end crashes	13	2
Lange changing crashes	2	0
Total crashes	15	2

### Sensitivity analysis

**Figure 12:**
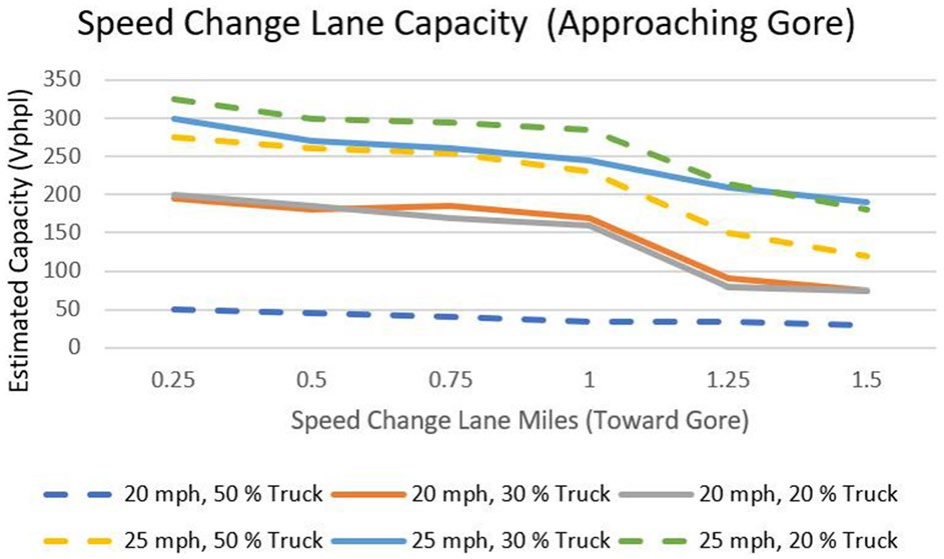
Estimated speed change lane capacity analysis.

Sensitivity analyses can be helpful in understanding the impact of various factors on estimated capacity and can inform decision making about potential changes that could be made to improve capacity. The results of the sensitivity analyses shown in Figure 12 suggest that an increase in truck volume can result in substantial delays near the gore, especially when capacity is approaching its limits. Additionally, the results indicate that longer speed change lanes and higher speed limits may have an impact on the estimated capacity and could result in reduced delays. Longer speed change lanes refer to sections of the arterial where vehicles are allowed more distance to change their speed, typically while entering or exiting the main flow of traffic. It's important to consider these results in the context of the specific gore area in road and traffic conditions in question and weigh the potential benefits and trade-offs of any changes that might be made based on the results of the sensitivity analyses.

## Conclusions

In this study, we conducted concurrent micro-simulation and Surrogate Safety Analysis to assess the real-time operational safety of a sharp gore area, focusing on a case study involving a diverging ramp in Lincoln, Nebraska. The study collected data on travel times, queue lengths, and physical ramp elements such as lane width, shoulder width, and so on, which were then used to create a VISSIM pre-intervention simulation model. Following model calibration, a few geometric elements of the onsite conditions, such as adding an auxiliary lane, increasing nose spacing and deceleration area, and increasing ramp spacing, were changed in the post-intervention simulation model. Three model calibration trials were performed to ensure that the error was less than 5%. The calibrated model used custom driving behaviors to include driving behavior of frequent drivers as well. Furthermore, SSAM analysis is performed for the before and after trajectory data to estimate the total number of rear-end, crossing, and lane-changing crashes that are likely to occur near the gore area of the off-ramp. Furthermore, Sensitivity analysis was carried out to understand the cause and effect of truck and total length of speed change lane on capacity of the gore areas. The sensitivity analysis showed that with increase in truck percentage in volume, the delay increases on gore areas.

Based on simulation results and Surrogate Safety Analysis, the study recommends adding an auxiliary lane in areas of sharp gore and emphasizes the importance of having enough deceleration area for safe vehicle maneuvering on diverging ramps. However, a proposed future cost-benefit analysis may change the current course of action because lane addition may incur additional costs. As, many transportation agencies, are focusing on achieving the goal of “vision zero,” or zero fatalities, we believe that adding separate auxiliary lanes near sharp gore areas can potentially help to prevent predicted crashes. In this study, data were collected for days to record travel times and traffic volumes, and the estimates might incur some degree of misestimation however, detailed AADT data can aid in the generation of precise VISSIM models with near-real accuracy. Due to limited resources and funding, we were unable to conduct a thorough model validation because This study examines the utility of SSAM in projecting future crash rates, specifically within the context of sharp gore areas, building upon its established effectiveness in various segments of the transportation network. We recommend rigorous model calibration and validation for future studies in order to estimate stronger models, particularly for congested traffic conditions on sharp gore areas, that can be added to the HCM. Additionally, we were unable to conduct simulations for lane additions or more gore areas in the vicinity of this ramp. Our research was carried out under specific circumstances and within defined timelines. Since that time, the city of Lincoln has already initiated the construction of an additional lane, directly influenced by our study. Consequently, there have been notable shifts in traffic parameters and conditions, which would pose a challenge in directly comparing our initial findings with the current situation. For future research, we recommend exploring the potential impact of lane additions through simulations. This approach would provide a more comprehensive understanding of the effectiveness and implications of implementing a relatively more costly intervention like lane expansion, particularly with regards to enhancing road safety. Furthermore, the choice to include truck percentage and volume in the sensitivity analysis was made to explore the broader impact of potential changes, given that trucks often play a significant role in traffic dynamics and safety concerns. While we recognize the value of conducting sensitivity analysis on each improvement separately, we were constrained by the current plans of the City of Lincoln to construct a separate lane, which includes a combination of changes as mentioned.

## Data Availability

All the data used in the city is properly reported within the text.
